# An Algorithm Based Wavelet Entropy for Shadowing Effect of Human Detection Using Ultra-Wideband Bio-Radar

**DOI:** 10.3390/s17102255

**Published:** 2017-09-30

**Authors:** Huijun Xue, Miao Liu, Yang Zhang, Fulai Liang, Fugui Qi, Fuming Chen, Hao Lv, Jianqi Wang, Yang Zhang

**Affiliations:** 1 Department of Biomedical Engineering, Fourth Military Medical University, Xi’an 710032, China; xinyin20130419@163.com (H.X.); lium90@163.com (M.L.); liangfulai@fmmu.edu.cn (F.L.); qifgbme@outlook.com (F.Q.); fmmulvhao@fmmu.edu.cn (H.L.); 2 Center for Disease Control and Prevention of Guangzhou Military Region, Guangzhou 510507, China; zyfmmu@126.com; 3 Department of Medical Engineering, Lanzhou General Hospital of Lanzhou Military Area Command of PLA, Lanzhou 730050, China; cfm5762@126.com

**Keywords:** ultra-wide band (UWB), multiple target detection, wavelet entropy

## Abstract

Ultra-wide band (UWB) radar for short-range human target detection is widely used to find and locate survivors in some rescue missions after a disaster. The results of the application of bistatic UWB radar for detecting multi-stationary human targets have shown that human targets close to the radar antennas are very often visible, while those farther from radar antennas are detected with less reliability. In this paper, on account of the significant difference of frequency content between the echo signal of the human target and that of noise in the shadowing region, an algorithm based on wavelet entropy is proposed to detect multiple targets. Our findings indicate that the entropy value of human targets was much lower than that of noise. Compared with the method of adaptive filtering and the energy spectrum, wavelet entropy can accurately detect the person farther from the radar antennas, and it can be employed as a useful tool in detecting multiple targets by bistatic UWB radar.

## 1. Introduction

In recent years, the application of ultra-wideband (UWB) radar for remote sensing of human beings at short ranges through walls and ruins has led to a range of intensive studies [1,2,3]. Different from thermal imaging, magnetic induction, X-ray, and some other success-restrictive methods [4], UWB radar determines human objects by detecting his/her chest subtle motion due to respiration, without the influence of temperature, material of non-metal wall and target clothes [2,5]. Furthermore, because the low-frequency pulse signals occupy an extremely broad bandwidth, the electromagnetic wave generated by UWB radar is beneficial to penetrate walls and various nonmetal materials. Moreover, compared with the continuous-wave radar system, UWB radar can locate human targets with the required accuracy. Accordingly, the UWB radar is a good solution for detection and localization purposes in rescue missions after disasters such as earthquakes, mine accidents, and collapse [2,6,7,8,9].

Short-range human targets detection using UWB radar systems through walls and ruins has been studied, e.g., in [10,11,12,13]. Up to now, the bistatic UWB radar (with one transmitting antenna and one receiving antenna) has resolved detection of a single stationary human target, yet the problem of multi-stationary target detection has been less well addressed. Several experiments for detecting multiple stationary human targets through walls have been carried out and the detection results have shown that bistatic UWB radar is able to accurately detect targets who are closest to the radar antennas only, whereas other farther targets are not able to be detected accurately. This is mainly attributed to three factors. Firstly, because of the low reflectivity of human respiration and the strong backscattered responses of obstacles, the signal to noise and clutter ratio (SNCR) of the echo signal is low. Secondly, as the transmission distance increases, the energy of the electromagnetic wave is attenuated, hence, the energy of electromagnetic waves reaching farther targets is inevitably weaker than that reaching the closest target. Thirdly, the closest target reflects the partial energy of the electromagnetic wave and, as a result of the decrease of the radar electromagnetic illumination, a shadowing region is formed and the respiration signal of farther targets located in the shadowing region are too weak to be detected [14].

Some methods were applied for detecting the weak signal of human targets by bistatic UWB radar. For example, mean subtraction was applied to remove the stationary clutter to enhance the SNCR of the echo signal in a real scenario, but the disadvantage of mean subtraction is that this algorithm affects the scattering information of a specific human target [13,15]. Singular value decomposition (SVD) was also used for removing the interference of non-stationary clutter, but this method is not efficient with a low SNCR [16]. The most common algorithm is adaptive filter [11,17], which needs two channels of input signals, but this algorithm ordinarily initiates an echo signal over-fitting. These signal processing algorithms above can suppress interference in a certain sense, however, for multiple stationary human targets, especially the farther targets located in the shadowing region, they do not work well.

As in previous studies, during radar detection of the respiration of stationary human targets, the distance between the radar and targets does not change. This means that the respiration signal always appears at a constant moment in the propagation time. Considering that the physical size of the human body is a certain thickness, the respiration signal of the human target always appears in several neighboring cells; thus, the signal in the area of the human target location will present a characteristic of regularity. This is different from the characteristic of noise and clutter, which are more likely to be random and disordered. According to the theory of information, entropy is a natural approach to measure the degree of order of a signal through its entropy spectrum [18]. It is supposed that the entropy amplitude of human respiration and noise will have a certain difference. Furthermore, as a result of the radar echo signal of human respiration being non-stationary, wavelet transform (WT) is often used to analyze the signal of respiration in time-frequency decomposition. If the algorithm of entropy and WT are in mutual cooperation, wavelet entropy (WE) is expected to quantify the time dynamics with the order/disorder of the radar echo signal. In [19], since humans and animals have different characteristic radar echos, the wavelet entropy method was used to distinguish human targets and dogs, and experimental results showed that the entropy value of the human target is much lower than a dog’s. It also indicated that the signals of human targets are more in order than that of dogs. Fugui Qi et al. also employed wavelet entropy to detect apnea in the respiratory signal of obstructive sleep apnea (OSA) by bio-radar, with surveillance results showing that the entropy value of apnea is higher than that of normal respiration [20]. In all of the above, wavelet entropy has been applied to distinguish human targets and dogs, the normal respiration and apnea with the assistance of the time-frequency characteristic of human respiration. However, analyzing the echo signal with WE analysis for detecting multiple targets, especially in the case of the shadowing effect, has not been reported [21,22]. Considering that entropy is closely related to the characteristic of the time-frequency instead of its energy, WE is proposed in this paper for detecting multiple stationary human targets.

The five sections are discussed as follows: In Section 2, the IR-UWB radar system is briefly described. Section 3 states three experimental scenarios of multiple target detection. The method for detecting multiple human targets in the shadowing effect is proposed in Section 4, and some data are analyzed. Then, the experiment results are presented and analyzed in Section 5. Finally, the conclusion and some proposals for future work are provided in Section 6.

## 2. UWB Radar System

**Figure 1:**
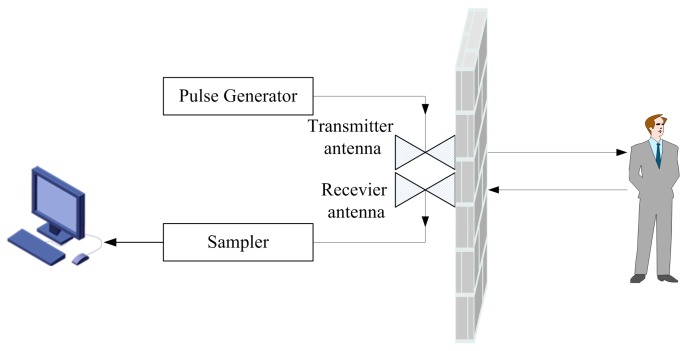
Bistatic ultra-wideband (UWB) bio-radar setup.

**Figure 2:**
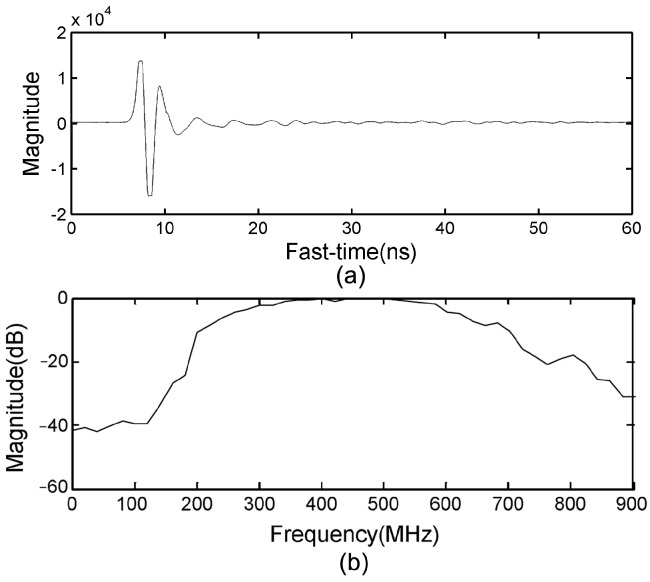
(**a**) One received waveform; (**b**) and the spectrum of the received waveform.

The UWB radar setup is shown in Figure 1. The UWB radar is stuck on the wall with two bow-tie dipole antennas, which are employed for transmitting and receiving the electromagnetic wave, respectively. A pulse generator produces trigger signals with a pulse repetition frequency of 128 kHz. The pulse signals are sent to the transmitter and shaped into bipolar pulses for exciting the transmitter antenna. The echo pulses are received by the receiver antenna, which is identical to the transmitter antenna. Then, the received pulses are sampled by means of equivalent-time-sampling with different time delay, thus, the signals of different ranges away from radar can be obtained. These signals were integrated and amplified, then stored in the form of waveforms through the digital signal processor. The waveforms are sampled into 2048 points. The recorded duration is 60 ns, and the detection range was up to 9 m. Considering that the UWB radar will be used for life detection and identification after an earthquake, it demands that the radar guarantee adequate penetrability and spatial resolution. Thus, the bistatic UWB radar with a center frequency and bandwidth of 500 MHz, respectively, was employed. A received waveform and its spectrum are shown in Figure 2a,b, respectively.

## 3. Experimental Statement

**Figure 3:**
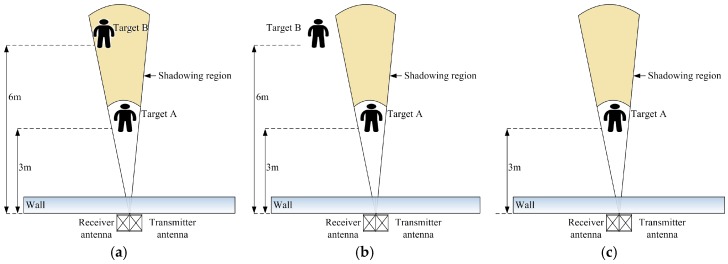
(**a**) Target A stood at 3 m in front of the antennas, while target B stood at 6 m in the shadowing region of target A; (**b**) target A stood at 3 m in front of the antennas, while target B stood at 6 m out of the shadowing region; and (**c**) target A stood at 3 m in front of the radar antennas.

In order to illustrate the fundamental problem of multi-stationary human target detection, several males aged between 25 and 30 years were involved, and three cases of experiments were carried out in this study. Measurements took place in the Bio-Radar Laboratory, and experiments were performed to detect human targets through a 24-cm-thick brick wall. For the first experiment, which is shown in Figure 3a, target A who was closer to the radar antennas stood at 3 m in the front of the antennas, and target B who was father away from the antennas stood at 6 m in the shadowing region of target A. The second experiment was shown in Figure 3b; target A retained the same position as the first scenario, and target B stood at 6 m out of the shadowing region. The third experiment was shown in Figure 3c; there was only one person located at 3 m in front of the radar antennas. 

## 4. Signal Processing and Analysis

### 4.1. Signal Pre-Processing and Analysis

The above-mentioned received waveforms were stored in a two-dimensional matrix R with *M* × *N*, where *M* indicates the detection distance, and it was sampled at discrete instants in fast time $\tau =mT_{f}(m=1,2,\ldots ,M)$; each fast time corresponded with a specific distance. N indicated the time signal of each fast time, and it was sampled in slow time $t=nT_{s}(n=1,2,\ldots ,N)$. In order to better estimate the stationary human targets, the signal pre-processing was applied in three steps: (i) Distance accumulation was used for compressing the sample point along the fast time dimension to shorten the length of the data, and the sample point was compressed from 2048 to 200; (ii) The clutter caused by stationary objects, such as walls, usually leads to a baseline drift in those signals along the slow time dimension. For removing the clutter of stationary objects, the average of all waveforms’ values with slow time were subtracted by a slide window, and the width of the slide window was 100; (iii) A low pass filter was applied to remove substantial random noise. As the respiration signal is a kind of low-frequency narrowband signal, a finite impulse response (FIR) filter was adopted along the slow time dimension for suppressing the high-frequency noise. Then, a new matrix *W* could be expressed as $W=(w_{1},w_{2},w_{i}\ldots w_{200})$, where row vector $w_{i}$ contained 64t (t was sampling time in slow time, the unit was second) sampling point. 

**Figure 4:**
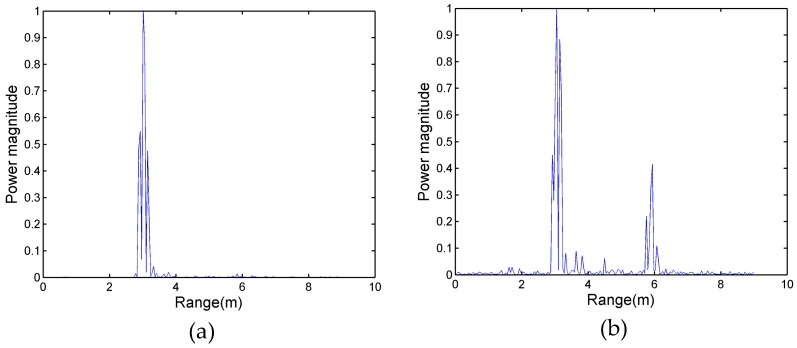
(**a**) The power spectrum of two targets with a shadowing effect; and (**b**) the power spectrum of two targets with no shadowing effect.

After pre-processing, the power spectrum of the data in Figure 3a was calculated; the result was shown in Figure 4a. Meanwhile, experimental data of Figure 3b were processed by the same method and the power spectrum is shown in Figure 4b. In previous studies, the magnitude of the power spectrum in the range dimension represents the degree of the signal power at different ranges [19]. In Figure 4a, the maximum peak appears at a distance of 3 m, which corresponds to the location of target A. However, target B cannot be detected, since he stood in the shadowing region. Figure 4b shows that there are two peaks that appear at distances of 3 m and 6 m, respectively; the magnitude of the peak at a distance of 6 m is smaller than that at 3 m. This is because of the attenuation of the electromagnetic wave of the radar as the distance increases. Therefore, the traditional power spectrum method is not credible for detecting multiple human targets, especially in the case with the shadowing effect.

**Figure 5:**
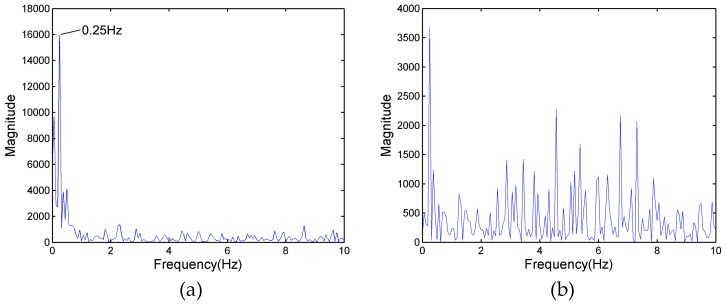
(**a**) The frequency spectrum of target B in Figure 3a; and (**b**) the frequency spectrum at the same distance as target B of Figure 3a in Figure 3c.

In Figure 3a, as a result of target A partially reflecting the energy of the electromagnetic wave, the energy reflected from target B is reduced compared with the energy from the same position of a single person (only one person standing at 6 m). Fortunately, although the power spectrum of target B cannot be observed in Figure 4, the signals of target B are also received by radar antenna. Figure 5a shows the frequency spectrum of target B in Figure 3a. The signals have many frequency components; a principal frequency of 0.25 corresponds to human respiration. In order to find the features of the signals of target B, who is located in the shadowing region, the signals of the same position in Figure 3c were processed, and the result is shown in Figure 5b. There are wider bands with more complex frequency components than Figure 5a.

### 4.2. Auto-Correlation Algorithm

As previously known, correlation is closely related to the characteristic of the period of respiration rather than that of energy. In order to improve the SNCR of the signal after pre-processing, an auto-correlation algorithm is proposed, which is fundamental for wavelet entropy. The equation is expressed as follows:

(1) $$\begin{array}{ll} R_{x}(t) & =E[x(t)x(t-\Delta t)]=E\left\{\left[s(t)+n(t)][s(t-\Delta t)+n(t-\Delta t)\right]\right\}\\ & =E[s(t)s(t-\Delta t)]+E[n(t)n(t-\Delta t)]+E[s(t)n(t-\Delta t)]+E[n(t)s(t-\Delta t)]\\ & =R_{s}(t)+R_{n}(t)+R_{sn}(t)+R_{ns}(t) \end{array}$$

Where s(t) is the wanted signal in time dimension after the step of pre-processing, n(t) is noise, x(t)=s(t)+n(t) is a mixed signal in the time dimension. An auto-correlation algorithm is implemented for each row vector $w_{i}$. Since the human life signal is not correlated with noise in the time dimension, $R_{sn}(t)$ and $R_{ns}(t)$ are approximately zero, and as Δt increases, the major energy of noise focuses on the location of Δt=0, $R_{x}(t)$ is equal to $R_{s}(t)$, and the noise is removed.

**Figure 6:**
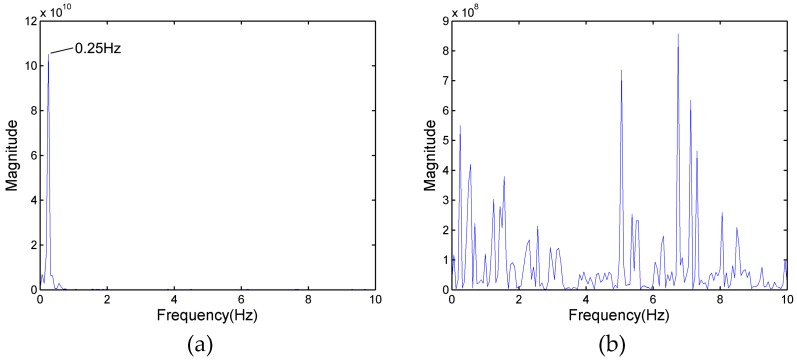
(**a**) The frequency spectrum of Figure 5a processed by auto-correlation; and (**b**) the frequency spectrum of Figure 5b processed by auto-correlation.

Figure 6a shows the results of the auto-correlation algorithm for the data of Figure 5a. As mentioned above, the respiration signal appears in several cells and they have a high correlation with each other. Thus, in Figure 6a, the stationary clutter and noise are eliminated, and the principal respiration frequency of 0.25 is reserved. Figure 6b shows the frequency spectrum of Figure 5b, where there is only one human target at a position of 3 m. Compared to Figure 5b, the frequency components have been reduced, but are richer than in Figure 6a. 

### 4.3. Wavelet Transform

Wavelet transform is a useful tool for analyzing a non-stationary signal, such as that of radar. Continuous wavelet transform (CWT) decomposes the continuous signal into a family of functions localized in time and frequency, respectively. The decomposition is constituted by a set of functions, $\psi_{a,\tau }(t)$, which are constructed by dilation and translation of a function ψ(t), called the mother wavelet. In this formulation, the parameter a is a dilation factor which measures the degree of compression, and the parameter τ is the translation factor which determines the time location of the wavelet. $\psi_{a,\tau }(t)$ is defined as:

(2) $$\psi_{a,\tau }(t)=\frac{1}{\sqrt{a}}\psi (\frac{t-\tau }{a}),\;a,\tau \in R,\;a\neq 0$$

Compared with CWT, the discrete wavelet transform (DWT) is more computationally efficient. The main difference is that the dilation factor a and the translation factor τ of the DWT can only have power of two values, i.e.,

(3) $$a=2^{j}$$

(4) $$\tau =ka=k2^{j}$$

where j is the level of DWT analysis performed and k is the parameter, k∈Z.

The coefficients C(a,τ) of the DWT can be divided into approximation coefficients and detail coefficients. The approximation coefficients are the high-scale, low-frequency components of signal f(n), whereas the detail coefficients are the low-scale, high-frequency components.

The discrete wavelet transform approximation coefficients of signal f(n) at level j are expressed as follows:

(5) $$A_{j}=\sum_{0}^{\infty }f(n)\phi_{j,k}(n)=\sum_{0}^{\infty }f(n)\frac{1}{\sqrt{2^{j}}}\phi (\frac{n-k2^{j}}{2^{j}})$$

where $\phi_{j,k}(n)$ is the scaling function associated with the wavelet function $\psi_{j,k}(n)$. Similarly, the detail coefficients of level j are expressed as follows:

(6) $$D_{j}=\sum_{0}^{\infty }f(n)\frac{1}{\sqrt{2^{j}}}\psi (\frac{n-k2^{j}}{2^{j}})$$

**Figure 7:**
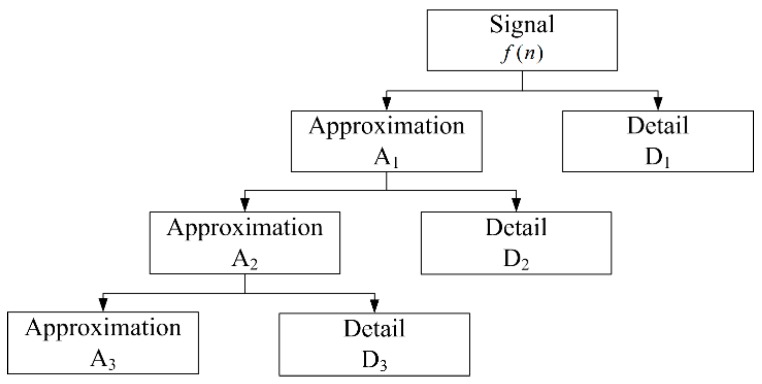
An example of a three-level wavelet decomposition of signal f(n).

Based on Equations (5) and (6), the signal f(n) is iteratively decomposed as the number of levels increases. Thus, a hierarchical set of approximations and details can be obtained through various decomposition levels. In this paper, the example of the three-level wavelet decomposition of signal f(n) is shown in Figure 7. Firstly, f(n) is split into an approximation A_1_ and a detail D_1_. Secondly, the approximation A_1_ is also split into a second-level approximation A_2_ and a detail D_2_. Thirdly, the approximation A_2_ is also split into a third-level approximation A_3_ and a detail D_3_.

### 4.4. Wavelet Entropy Algorithm

In order to better explain the flow of signal processing by wavelet entropy algorithm, the signal vector in the slow time dimension processed by auto-correlation is considered to be $X_{\tau }(t)(\tau =1,2,\ldots ,200)$. As shown in the following, the DWT of signal $X_{\tau }(t)$ is defined as:

(7) $$DWT_{s}(j,k)=\sum_{0}^{\infty }X_{\tau }(t)\psi_{j,k}(t)$$

The wavelet coefficient $C_{j}(k)$ not only provides relevant information in a simple way, but also provides an estimation of the location energies at different scales. It is supposed that the sample value $X=\left\{x_{0}(n),n=1,\ldots ,N\right\}$ corresponds to a uniform time grid. If the discrete dyadic wavelet decomposition is implemented over all resolution levels, the wavelet expansion of $X_{\tau }(t)$ can be expressed as:

(8) $$X_{\tau }(t)=\sum_{j=-J}^{-1}\sum_{k}C_{j}(k)\;\psi_{j,k}(t)\;j=-J,\ldots ,-1\;(J=\log_{2}(N))$$

where $C_{1}(n),C_{2}(n),\ldots ,C_{J}(n)$ are the wavelet coefficients of the wavelet sequence.

If the family $\left\{\psi_{j,k}(t)\right\}$ is an orthonormal based on the Hilbert space $L^{2}(R)$, the concept of energy at the resolution level can be defined as:

(9) $$E_{\tau ,j}={\sum_{k}\left|C_{j}(k)\right|}^{2}$$

The radar signal is divided into non-overlapping temporal windows of length L to follow the temporal evolution of the quantifiers. For each interval i,(i=1,…,NT,NT=NL) with N samples, the appropriate signal values are assigned to the central point of the time window. The concept of the average energy of resolution level j can be defined as:

(10) $$E_{\tau ,j}=\frac{1}{N_{j}^{\left(i\right)}}{\sum_{(i-1)L+1}^{iL}\left|C_{j}(n)\right|}^{2},\;i=1,\ldots ,N_{T}$$

where $N_{j}^{\left(i\right)}$ represents the number of wavelet coefficients at resolution j included in the time window i. The total energy of the wavelet coefficients at this time window can then be obtained by:

(11) $$E_{\tau ,total}=\sum_{j}E_{\tau ,j}$$

The relative wavelet energy that represent the energy’s probability distribution is:

(12) Pτ,j=Eτ,jEτ,total

Clearly, $\sum_{j}P_{\tau ,j}=1$. According to the Shannon entropy, which gives a measure of the information on any distribution for analyzing and comparing the probability distribution, the wavelet entropy is defined as:

(13) $$H_{\tau }(P)=-\sum_{j}P_{\tau ,j}\ln[P_{\tau ,j}]$$

**Figure 8:**
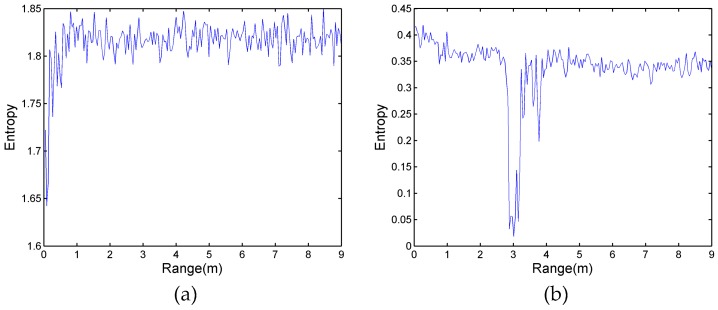
(**a**) The value of wavelet entropy with no human target; and (**b**) the value of wavelet entropy with one human target located at 3 m.

In the frequency spectrum analysis of Figure 6, the respiration of the human target produces a narrow-band spectrum; it indicates that the respiration signal is regular. However, the signal of the noise produces a very wide-band spectrum, which indicates that noise is disordered. According to entropy theory, a regular signal is inclined to produce lower-value entropy. Conversely, a completely random signal, such as clutter and noise, is inclined to a higher entropy value. In order to eliminate the effect of wavelet entropy, two experimental scenarios with no human target and a single human target located at 3 m are implemented. The wavelet entropy spectrum of these two scenarios is exhibited in Figure 8. Figure 8a shows that the entropy value of the experiment with no human target fluctuated as the range was processed. Excluding the influence of the reflection of the wall from 0 m to 1 m, the entropy value of the remaining region is from about 1.79 to 1.85. In Figure 8b, as the target is located at 3 m, the entropy value of the target is the lowest, owing to the trailing effect of the target, and the entropy value at 3.8 m is lower than the other noise region.

## 5. Experiment Results and Discussion

In this section, in order to demonstrate the capability of the wavelet entropy algorithm to detect multi-stationary human targets, the adaptive filter algorithm and power spectrum were also used as references. Two cases of experiments imitating Figure 2 were conducted and one variable was assigned in each experimental case. For the first experimental case, the variable is the distance of target B to radar antennas. Three experiment scenarios were described in the following: For experiment I, target A stood 3 m in front of the antennas, and target B stood 5 m in the shadowing region of target A. For experiment II and experiment III, target A remained in the same position as in experiment I, but the distance of target B to the radar antennas was 6 m and 7 m, respectively. For the second experimental case, the variable is the intersection angle of target A and target B. There were five experiment scenarios. Target A stood at 2.5 m in front of radar antennas and the distance of target B to the radar antennas was 4 m, but their intersection angles were 10°, 15°, 20°, 25°, and 30°.

**Figure 9:**
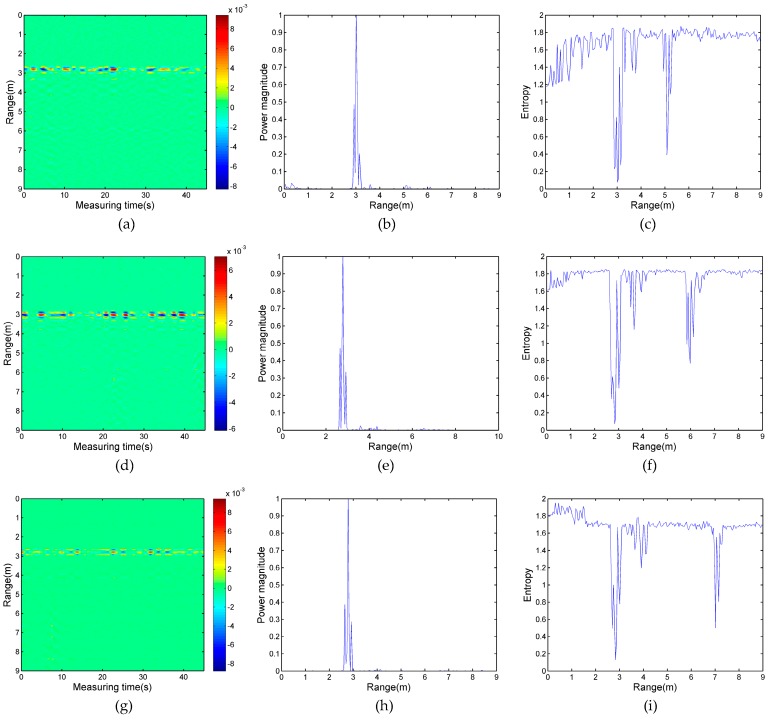
(**a**) The data of experiment I processed by an adaptive filter; (**b**) the power spectrum of the data of experiment I; (**c**) the entropy spectrum of the data of experiment I; (**d**) the data of experiment II processed by an adaptive filter; (**e**) the power spectrum of the data of experiment II; (**f**) the entropy spectrum of the data of experiment II; (**g**) the data of experiment III processed by an adaptive filter; (**h**) the power spectrum of the data of experiment III; and (**i**) the entropy spectrum of the data of experiment III.

**Figure 10:**
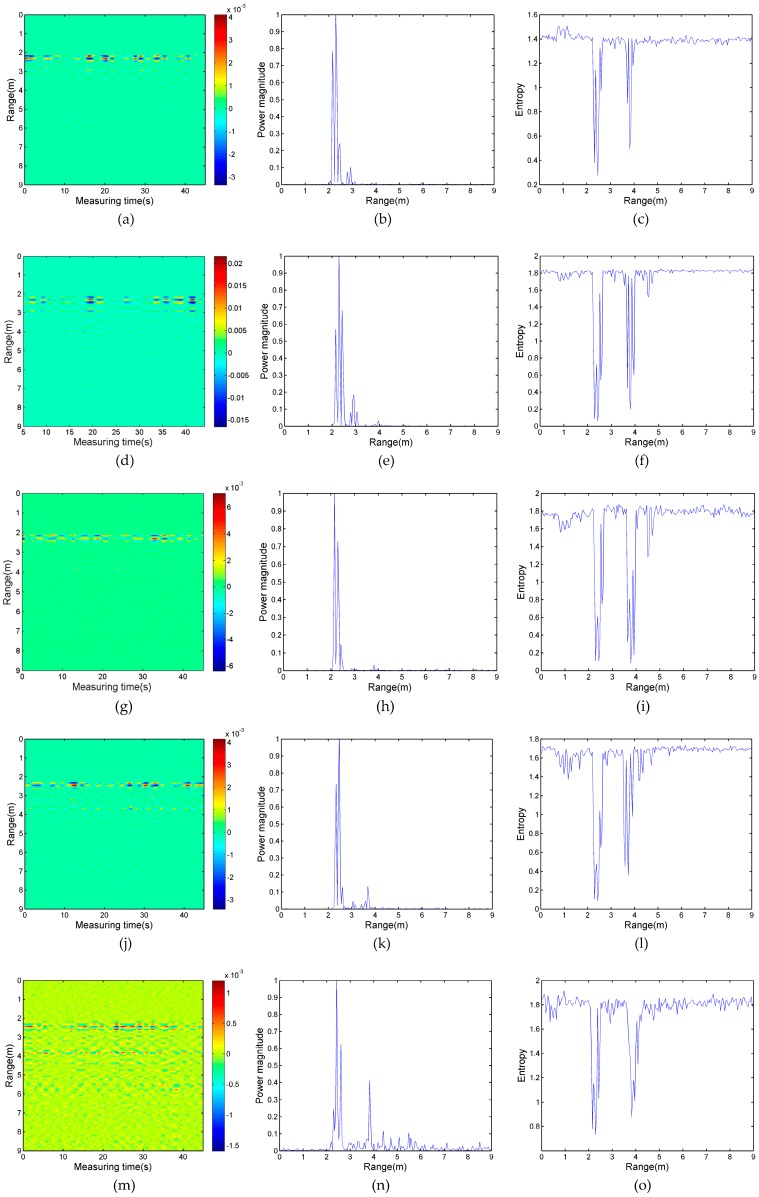
(**a**) The experimental data of the 10° intersection angle processed by an adative filter; (**b**) the power spectrum of the experimental data with a 10° intersection angle; (**c**) the entropy spectrum of the experimental data with a 10° intersection angle; (**d**) the experimental data of a 15° intersection angle processed by an adative filter; (**e**) power spectrum of the experimental data with a 15° intersection angle; (**f**) the entropy spectrum of the experimental data with a 15° intersection angle; (**g**) the experimental data of a 20° intersection angle processed by an adative filter; (**h**) the power spectrum of the experimental data with a 20° intersection angle; (**i**) entropy spectrum of the experimental data with a 20° intersection angle; (**j**) the experimental data of a 25° intersection angle processed by an adative filter; (**k**) the power spectrum of the experimental data with a 25° intersection angle; (**l**) entropy spectrum of the experimental data with a 25° intersection angle; (**m**) the experimental data of a 30° intersection angle processed by an adative filter; (**n**) the power spectrum of the experimental data with a 30° intersection angle; and (**o**) the entropy spectrum of the experimental data with a 30° intersection angle.

Real experimental data were processed by the wavelet entropy algorithm and two reference algorithms based on the adaptive filter and power spectrum. The experiment results of the first and the second experimental case are shown in Figure 9 and Figure 10. In Figure 9, only one periodically-varied strip that corresponds to the respiratory-motion response is obvious at the range of 3 m in Figure 9a,d,g. The results of the power spectrum in Figure 9b,e,h are similar to the above pseudo-color, with only one peak located at 3 m away from the radar in each Figure, respectively, which are correlated with target A. The energy of target B (at 5 m, 6 m, and 7 m in the first experimental case), who was located in the shadowing region, is negligible compared to that of target A. Thus, the method based on an adaptive filter and energy detection could easily induce a false negative result. 

According to the result of Figure 8, the entropy value displays a significant difference in the condition with a human target or without a human target. In this section, the proposed algorithm is also applied for locating the distance of multi-stationary human targets. Figure 9c,f,i show the results of the first case of experimental data processed by wavelet entropy. In these three Figures, the entropy value at 3 m is the lowest, which indicates that the position of target A is accurately estimated. Likewise, another lower entropy value—5 m in Figure 9c, 6 m in Figure 9f, and 7 m in Figure 9i—indicates the position of target B in the three experiments. However, it is noteworthy that the entropy values within 1 m behind target A are lower than the rest of the remaining region in the two Figures; it is easy to incorrectly deduce that one human target is located in this region. According to the theory of electromagnetic wave propagation, as a result of multipath effects and tailing effects, the ground reflects the respiration signals of target A, and the signals behind target A are regular and have the same frequency as the respiration of target A. Furthermore, the respiration signals appear in several neighboring cells and the physical size of the human body is not ignorable. In Figure 9c, the entropy pit of target A is from 2.835 to 3.24 and the entropy pit of target B is from 4.905 to 5.31. In Figure 9f, the entropy pit of target A is from 2.61 to 3.15 and the entropy pit of target B is from 5.76 to 6.25. In Figure 9i, they are from 2.61 to 3.10 and from 6.93 to 7.33; the six ranges of pits correspond to the thickness of the human body. For the lower entropy value that is behind target A, the range of the pits does not obviously agree with the thickness of the human target. Thus, the algorithm of the wavelet entropy can detect two stationary human targets accurately despite one person being in the shadowing region. 

The results of the second experimental case are shown in Figure 10. From Figure 10a–i, the intersection angle of target A and target B is 10°, 15°, and 20°, and target B is located in the shadowing region of target A. The results of the experiment processed by the proposed and referenced algorithms complied with the expected results. Even though the distance of the human target to the radar antennas is decreased, due to the shadowing effect of target A, target B cannot be detected by an adaptive filter and the power spectrum. Under the condition that the intersection angle of two targets reduces to 10°, target A reflects almost all of the energy of the electromagnetic wave to target B, and target B can still be detected by the wavelet entropy algorithm. As their intersection angle increases, the energy of the echo signal of target B is stronger, thus, when the intersection angle is 25° and 30°, in Figure 10j,m, the periodically-varied strip corresponding to the respiratory-motion response of target B appears at the range of 3.7 m and 3.8 m, respectively. There are also small peaks of target B located at 3.7 m and 3.8 m away from the radar in each power spectrum of Figure 10k,n, respectively. Thus, when the intersection angle is less than 20°, the wavelet entropy is more effective for detecting multi-stationary human targets.

## 6. Conclusions

In the case of multi-stationary human target detection through a brick wall using bistatic UWB radar, since the person closest to radar antennas partially reflects the energy of the electromagnetic wave, the person farther from the radar antennas is not detected accurately, especially when in the shadowing region of the closest person. Considering that the signals of the farther person are contained in the echo signal, and its frequency spectrum is different from the characteristic of noise, wavelet entropy is proposed as a new quantitative algorithm to detect human targets in the shadowing region. This algorithm utilizes the difference between periodic respiration and random noise in the time-frequency domain and calculates the entropy based on the complexity of energy after wavelet transform. In this paper, as the respiration of the human target exhibits more rhythmic behavior compared with noise, the entropy of the human target was significantly lower than in other regions. Through analyzing the two cases of experimental results, a conclusion is drawn: the reference algorithms based on adaptive filtering and the power spectrum can only detect the closest person; they are incapable of detecting the person in the shadowing region. The wavelet entropy algorithm not only detects the closest person and locates their distance, but also detects the farther human target in the shadowing region and gives an accurate distance. This is a breakthrough in detecting multi-stationary human targets. Yet, it must be admitted that the bistatic radar with one transmitter antenna and one receiver antenna restricts the expansion of the multi-stationary human target’s detection. Therefore, in future research, we will consider applying an UWB radar array for detecting multi-stationary targets. We think that information fusion of multiple channels may essentially improve the subtle signal of the remaining shadowed human targets in this kind of situation.
